# Sequencing and analysis of the mitochondrial genome of *Pituophis catenifer sayi* (Squamata: Colubridae)

**DOI:** 10.1080/23802359.2016.1192497

**Published:** 2016-07-10

**Authors:** Abhimanyu Lele, Matthew S. Rand, Stephan G. Zweifel

**Affiliations:** Department of Biology, Carleton College, Northfield, MN, USA

**Keywords:** Colubrinae, mitogenome, *Pituophis catenifer sayi*, phylogenetic analysis

## Abstract

The complete DNA sequence of the mitochondrial genome of the bullsnake (*Pituophis catenifer* sayi) is presented and analyzed in this study. The genome is 17,193 bp in length, and contains 22 transfer RNA genes, 2 ribosomal RNA genes, 13 protein-coding genes and 2 control regions. The overall base composition of the H-strand is A (34.5%), T (26.2%), C (25.8%) and G (12.7%). The gene order and orientation of the mitogenome is consistent with other sequenced genomes from colubrid snakes. Phylogenetic analyses using the ML, NJ and MP methods for a set of colubrids, including every sequenced genus within the sub-family Colubrinae, produced identical trees. We show that *Pituophis* is most closely related to the American ratsnake genus *Pantherophis.*

*Pituophis catenifer sayi*, commonly known as the bullsnake, is a member of the most speciose family of snakes in the world, Colubridae. A sub-species of the gopher snake *Pituophis catenifer*, the distribution of this multispecies genus ranges from Canada to Mexico and the Pacific coast to the Atlantic (Conant & Collins 1998). The bullsnake is a species of conservation concern in the states of Minnesota, Wisconsin and Iowa due to habitat fragmentation (Kapfer et al. 2008). We present the mitochondrial DNA sequence analysis of this snake, which to the best of our knowledge is the first sequenced mitogenome of the genus *Pituophis*. DNA was extracted from shed skins and then amplified using a series of 24 overlapping PCR primer pairs. Both strands of DNA were sequenced and aligned to confirm base pair identity. The specimen was collected at the following location: 36^°^ 0’51.37’’ N, 97^°^ 17’15.72’’ W (near Cimarron River, Payne Co., OK). Specimen (currently living) and shed skin samples are in Carleton College’s natural history collection in the Biology Department (accession #CC-51864).

The mitochondrial genome is a double-stranded circular DNA sequence with a length of 17,193 base pairs. It includes 22 transfer RNA genes, 2 ribosomal RNA genes, 13 protein-coding genes and 2 control regions. The accurate annotated mitochondrial genome sequence was submitted to Genbank with the accession number KU833245. The arrangement of genes is similar to that found in other colubrid mitochondrial genome sequences (Dong & Kumazawa 2005). The overall composition of the heavy strand is A (34.5%), T (26.2%), C (25.8%) and G (12.7%). This AT bias did not substantially change in the control regions.

In addition to the control region (CRI) located between the *tRNA-Pro* and *tRNA-Phe* genes, *P. catenifer sayi* has a duplicated control region (CRII) located between the *tRNA-Ile* and the *tRNA-Leu* genes. This duplication is consistent with what has been found in all other snakes except the most basal lineages (Dong & Kumazawa 2005). In *P. catenifer sayi,* the length of CRI is 1026 bp and the length of CRII is 1053 bp. In addition, we detected a 35 bp sequence between the *tRNA-Asn* and *tRNA-Cys* genes that has been proposed to be the origin of light-strand synthesis by nature of its ability to fold into a stem-loop structure (Liu & Zhao 2015)

Ten of the protein-coding genes begin with ATG as the start codon, while COX1 and ND6 begin with GTG, and ND2 begins with ATT. The genes ATP6, ATP8, ND4L, ND4 and ND5 terminate with the stop codon TAA, COX1 ends with AGA, ND2 ends with TAG, and the other protein coding regions end with an incomplete stop codon T––. Eight tRNA genes and ND6 are encoded on the light strand; the remaining genes are encoded on the heavy strand. Previous work reported the presence of a pseudo-*tRNA-Pro* gene adjacent to CRII in some Colubridae snakes (He et al. 2010; Yan et al. 2014). Our analysis of the *P. catenifer sayi* mitogenome does not indicate the presence of this DNA sequence, similar to results in *E. schrenckii* (Liu & Zhao 2015).

Phylogenetic analyses, using MEGA6 software (Tamura et al. 2013), of complete mitochondrial genome sequences using the ML, NJ and MP methods were performed on a set of colubrid snakes that included every extant genus of the subfamily Colubrinae sequenced to date. Two species of Viperidae were used as outgroups. All three methods yielded identical phylogenetic trees. The phylogram obtained by the ML method is shown in Figure 1. Among the sequenced taxa, *P. catenifer sayi* is most closely related to *Pantherophis slowinskii*. As has been previously discussed by Xu et al. (2015), we found *Oligodon ningshaanensis* to be more closely related to the subfamily Dipsadinae than to other species in Colubrinae. The other Colubrinae species form a monophyletic group with high bootstrap values.

**Figure 1.**
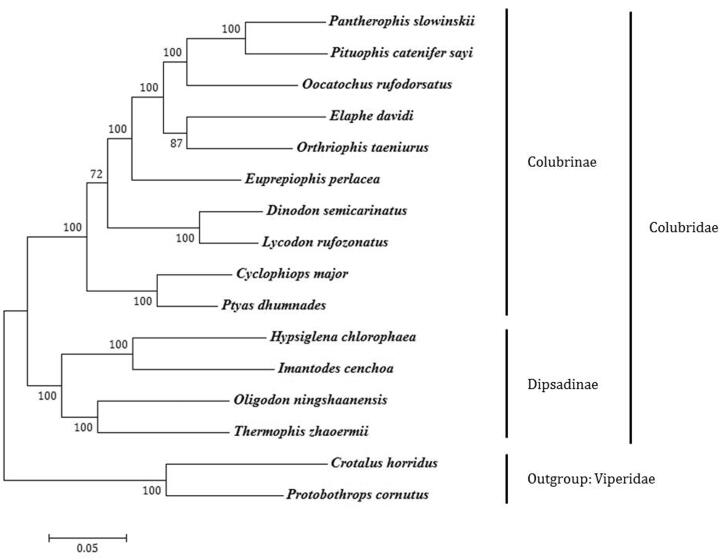
Phylogenetic tree generated using the ML method based on complete mitochondrial genome sequences. Subfamily designations are correct except for *O. ningshaanensis*, which is currently placed in Colubrinae. Branch lengths represent nucleotide substitutions per site. *Pantherophis slowinskii* (NC_009769), *Pituophis catenifer sayi* (KU833245), *Oocatochus rufodorsatus* (NC_022146), *Elaphe davidi* (KM401547), *Orthriophis taeniurus* (KC990021), *Euprepiophis perlacea* (KF750656), *Dinodon semicarinatus* (AB008539), *Lycodon rufozonatus* (NC_024559), *Cyclophiops major* (KF148620), *Ptyas dhumnades* (KF148621), *Hypsiglena chlorophaea* (KJ486459), *Imantodes cenchoa* (NC_013988), *Oligodon ningshaanensis* (NC_026083). *Thermophis zhaoermii* (GQ166168), *Crotalus horridus* (HM641837), *Protobothrops cornutus* (KF110978).
